# Hydrophilic Scaffolds Containing Extracts of *Stryphnodendron adstringens* and *Abarema cochliacarpa* for Wound Healing: In Vivo Proofs of Concept

**DOI:** 10.3390/pharmaceutics14102150

**Published:** 2022-10-10

**Authors:** Maria C. M. A. Alves, Marismar F. Nascimento, Bernadeth M. de Almeida, Matheus M. A. Alves, Isabel B. Lima-Verde, Daniela S. Costa, Daniela C. Medeiros Araújo, Mariana N. de Paula, João C. P. de Mello, Amanda Cano, Patricia Severino, Ricardo L. C. de Albuquerque-Júnior, Eliana B. Souto, Juliana C. Cardoso

**Affiliations:** 1 Post-Graduating Program in Health and Environment, University of Tiradentes, Av. Murilo Dantas, 300, Aracaju 49010-390, Brazil; 2 Department of Nursing, Campus Petrolina, University of Pernambuco, Recife 50100-010, Brazil; 3 Federal Institute of Sergipe, Aracaju 49055-260, Brazil; 4 Department of Pharmacy, Ingá University Centre, Maringá 87035-510, Brazil; 5 Laboratory of Pharmaceutical Biology, Palafito, Department of Pharmacy, State University of Maringá, Maringá 87020-900, Brazil; 6 Department of Pharmacy, Pharmaceutical Technology and Physical Chemistry, Faculty of Pharmacy and Food Sciences, University of Barcelona, 08007 Barcelona, Spain; 7 Institute of Nanoscience and Nanotechnology (IN2UB), 08028 Barcelona, Spain; 8 Institute of Technology and Research, University of Tiradentes, Aracaju 49032-490, Brazil; 9 Department of Pathology, Federal University of Santa Catarina, Florianópolis 88040-370, Brazil; 10 Department of Pharmaceutical Technology, Faculty of Pharmacy, University of Porto, Rua de Jorge Viterbo Ferreira 228, 4050-313 Porto, Portugal; 11 REQUIMTE/UCIBIO, Faculty of Pharmacy, University of Porto, Rua de Jorge Viterbo Ferreira 228, 4050-313 Porto, Portugal

**Keywords:** resorbable membranes, hydrophilic scaffolds, *Stryphnodendron adstringens*, *Abarema cochliacarpa*, gallocatechin, catechin

## Abstract

The present work aimed to evaluate the healing effect of hydrophilic polymeric resorbable biomembrane scaffolds containing plant extracts obtained from two different species, both popularly known as *Stryphnodendron adstringens* or *Barbatimão*. The hydrogel-based scaffolds were characterized and submitted to biological tests using Wistar rats to evaluate their healing capacity. The wound retraction index and the evaluation of the inflammatory process and tissue collagenization were recorded. The extracts showed antioxidant activity with IC50 between 10 and 20 µg/mL (DPPH assay) and 4–6 mmol Trolox/g (FRAP assay). The extract of *Stryphnodendron adstringens* (SA) presented gallocatechin, epigallocatechin, and *O*-methylpigalocatechin, while the extract of *Abarema cochliacarpa* (AC) presented catechin, dimers of procyanidins, di-*O*-hydroxide, *O*-deoxyhexosi-hexoside, and epicatechin. The membranes containing SA extract (GELSA) were more rigid, with a more intense color, but less thick, with a more compact structure and few pores. The membranes containing AC extract (GELAC) presented a mechanical profile like the gelatin membrane (GEL), with greater permeability to water vapor. The GELAC and GELSA membranes showed similar thermal degradation profiles. The wounds treated with the membranes containing the extracts obtained high levels of retraction of the wounds with values around 60% and 80% in three and seven days, respectively. These data indicate that the compounds of both species have promising biological activities in the repair process, showing that the extracts accelerated the healing process due to the lower intensity of the inflammatory reaction and the presence of compounds such as catechin and epigallocatechin.

## 1. Introduction

The chronicity of unhealed wounds, ulcers, and burns generates socioeconomic impacts and is considered a serious public health problem. In the U.S., chronic wounds have a prevalence of 2% in the population, which represents a cost to the health system of approximately US$ 25 billion each year [1]. There are many products on the market used for the healing of dermal lesions [2]. The number of different wound dressings exceeds from 3000 in the market, which enable physicians to consider all elements of wound repair. However, most of them have a range of limitations, such as high cost of production, difficulty of keeping and storing them, need to replace the dressing, and the fact that most dressings seem to act in specific and distinct phases of healing, acting as complicating factors for the best dressing selection [3]. Thus, research is still needed to find an improved treatment option for wound healing, especially those with a chronic clinical course.

A suitable wound dressing to provide a proper environment for the healing process include durability, flexibility, permeability to water vapor, adherence to the tissue, and suitable mechanical properties [4]. Gelatin, a polymer-based wound dressing material, fulfills all these requirements, as long as has demonstrated to have biocompatibility, hemostatic property, pH-sensitivity, and satisfactory swelling ability and water evaporation rate [5].

Wound healing is a process that can occur naturally, even without pharmacotherapeutic treatment [6]. However, conditions such as inadequate lifestyle habits, diabetes, obesity, vascular diseases, nutritional status, infections, and comorbidities may worsen skin recovery, contributing to the chronicity of the wound [7]. In this way, wound treatment often becomes a great and costly therapeutic challenge. The correct use of ointments and dressings influences the course of healing positively, and can reduce potential complications [1]. In this sense, some studies reporting the use of natural products have shown positive effects in the wound healing process, such as membranes based on, e.g., gelatin, chitosan, sodium alginate, and containing usnic acid [8,9], *Vitis labrusca* [10], aloe vera [11], *Punica granatum* Linn [12], and red propolis [13,14].

Some plant species are popularly known for their healing activities. Barbatimão is the popular name of two species widely used in Brazil. *Stryphnodendron adstringens* is recognized by the Brazilian Ministry of Health, being presented in the herbal medicine form of the Brazilian Pharmacopoeia as a healing in the pharmaceutical form of cream. Some biological actions are attributed to the stem bark extracts, such as, hemostatic, antioxidant, antibacterial and anti-inflammatory properties [15,16,17]. The popular name “barbatimão” is also used in reference to the species *Abarema cochliacarpos* [18]. The healing properties of *Stryphnodendron adstringens* have already been described in several works [19,20].

Products of natural origin (e.g., plant extracts) associated with biomaterials (e.g., biopolymers, lipids) can be an interesting alternative for the treatment of wounds at different stages of healing, providing a shorter healing time and patient comfort [10,11,12,21]. A synergistic effect between the plant extract and the biomaterial results in an effective fixation onto the injured skin, being absorbable by the wound without generating residues. The obtained product a promising alternative to minimize inflammatory signs and symptoms and, consequently, in helping the repair process of wounds. Moreover, these products are reproducible, and can be sterilized. The aim of this work was the development of a hydrophilic gel-based reabsorbable biomembrane containing hydroacetonic extracts of *S. adstringens* and *A. cochliacarpa* to be used in the healing process of open wounds in an animal model.

## 2. Methods

### 2.1. Plant Material

The skins of all samples were collected manually, with the aid of a cutting blade. *Stryphnodendron adstringens* (SA) barks were collected in São Jerônimo da Serra, Paraná, Brazil (24°43′78″ S, 50°45′24″ W) and the material sample was identified and deposited in the Herbarium of State University of Maringá under registration HUEM 28197. Barks of *Abarema cochliacarpa* (AC) were collected at Colônia Tejupeba, in the municipality of Itaporanga d’Ajuda—Sergipe, Brazil (11°4′41″ S, 37°17′23″ W). The material was identified and deposited in the Tiradentes University Herbarium under registration number HUT 815.

### 2.2. Extract Preparation

The extracts were obtained by turbo extraction and in triplicate, according to the methodology proposed by Ishida et al. (2006). The turbo-extractor was used (Ultra-Turrax UTC115KT) with acetone:water (7:3) as the extractor system. A total of 2 kg of plant material was sheared for 5 min, followed by 12 h of rest. After this time, 3 cycles of 5 min of agitation and 20 min of rest were performed. After turbolysis, the extract was filtered through a Büchner funnel. The organic solvent was removed using a rotary evaporator under reduced pressure, and the residue was lyophilized to obtain the dry crude hydroacetonic extract. The extract was evaluated by verifying the percentage yield of the extraction was calculated.

### 2.3. Chromatographic Analysis

The extract samples were prepared at a concentration of 1 mg/mL and injected into the chromatograph (1 µL), with a mobile phase flow rate of 0.3 mL/min and an oven temperature of 50 °C. The mobile phase was composed of deionized water (A) and acetonitrile (B), both with 0.1% formic acid. The elution gradient used was 0–2 min at 3% (B), 2–25 min from 3 to 25% (B), 25–40 min from 25 to 80% (B) and 40–43 min at 80% (B). The chromatographic peaks were identified based on their UV spectra, mass obtained for precursor ions—protonated molecule [M+H]⁺ and/or deprotonated molecule [M + H]^−^ in high resolution, spectra of product ions generated by dissociation collision induced in EM/EM mode and retention times in the system used (pattern co-injection). These data were generated from HPLC-DAD-MS and HPLC-DAD-MS/MS analyses, in both positive and negative ionization mode, for each sample. Some peaks were identified by comparing retention times and spectral data with those of standards, as well as by comparing data described in the literature for such substances.

### 2.4. Extract Antioxidant Evaluation

DPPH: The extracts were tested for DPPH radical reduction using the method described by Brand-Williams et al. [22]. The extracts were diluted in methanol at final concentrations of 3.1 to 100.0 μg mL^−1^. A total of 100 µL of freshly prepared 130 µM DPPH was added to 100 µL of the sample at different concentrations. The microplates were kept at room temperature and protected from light for the reaction to occur for 30 min, and the absorbances were determined at a wavelength of 517 nm in a microplate spectrophotometer (Bio-tek Power Wave XS, Agilent Technologies, Inc., Santa Clara, CA, USA). The equipment was zeroed with methanol. As a negative control, methanol was used, replacing the sample, and as a positive control, a standard solution of quercetin was used. The percentage of DPPH free radical scavenging (*FRS*, %) was calculated using the following equation:

$$\mathrm{FRS}\left(\%\right)=\frac{A_{c}-A_{s}}{A_{c}}\times 100\;$$

where *A_c_* is the absorbance of the negative control and *A_s_* is the absorbance of the extract solution. With the values obtained, a graph of percentage of free radical scavenging versus concentration of the tested extracts was constructed. By means of linear regression, the EC50 was calculated. For the determination of antioxidant activity using the method with the FRAP reagent, Trolox standard (20 µM to 600 µM) and extracts (20 to 80 µg mL^−1^) were prepared using ethanol as solvent. To prepare the FRAP reagent, 2.5 mL of tripyridyltriazine (TPTZ) solution, 2.5 mL of 20 mM iron chloride hexahydrate (FeCl_3_·6H_2_O) solution and 25 mL of 0.3 mM acetate buffer were mixed. The FRAP solution was incubated at 37 °C in a water bath for 30 min. For the reaction, an aliquot of 30 μL of different concentrations of the Trolox standard or extracts was added to 150 μL of the FRAP reagent. This mixture was homogenized and read in a microplate spectrophotometer (Bio-tek Power Wave XS) at 595 nm, after incubation for 30 min in a water bath at 37 °C. The equipment was zeroed with the sample blank (150 μL of 0.3 mM acetate buffer and 30 μL of the sample). The results were expressed in the antioxidant capacity of the extracts equivalent to Trolox (TEAC) from the standard curve obtained with the Trolox samples.

### 2.5. Production of Polymeric Biomembranes

Powdered gelatin (NP Comércio de Produtos Alimentícios, Ltda, Cotia, São Paulo, Brazil) was dispersed in distilled water (1% *m/v* dispersion). The extracts (1% *m/m* in relation to the polymeric mass), previously solubilized in propylene glycol (20% *m/m* in relation to the polymeric mass) were added to the gelatin dispersion. Membranes were produced by the casting method, where 30 mL of the film-forming dispersion was poured into polypropylene plates (7.5 cm in diameter) and the solvent was removed in a fume hood. The biomembranes obtained were placed in containers with a relative humidity of 58%. Foram produzidas membranas sem extrato (GEL), com extrato de *S. adstringens* (GELSA) a 1% e com extrato de *A. cochliacarpa* (GELAC) a 1%.

### 2.6. Mechanical Properties

The mechanical properties of the biomembranes were determined using a texturometer, in traction mode (TA-TX2, Stable Micro Systems, Surrey, UK). The samples were cut in a rectangular shape (30 × 10 mm) (*n* = 10), and the thickness was determined at three points of the sample using a digital caliper (precision ± 0.001 mm). Young’s modulus or elasticity (E) was calculated from the linear region of the stress x strain curve, between 0.00% and 1.00% elongation. The strength (rupture stress and elongation) of the material was calculated through the area under the stress × strain curve. The separation of the grips was 20 mm and the test speed was 1 mm/s.

### 2.7. Water Vapor Permeability

The permeability of biomembranes was analyzed by gravimetry. Beakers (*n* = 5) containing saturated solution of potassium bromide (KBr) were sealed with the biomembranes under study. Then, they were weighed and placed in a desiccator containing silica. The mass loss related to water vapor permeation through the membrane was determined by successive weighing at planned times over a period of 48 h. The permeability was calculated using the following equation:

$$\mathrm{PVA}=\frac{m_{p}\times e}{t\times A\times \Delta \mathrm{PV}}$$

where *PVA* is the water vapor permeability; *m_p_* the lost mass (g); *e* the membrane thickness (mm), *t* the time (day); *A* the membrane area (m^2^), and Δ*PV* the difference between the water vapor pressure inside and outside the container (KPa).

### 2.8. Swelling Index

The swelling ratio of biomembranes (*n* = 3) measuring 2.0 × 3.0 cm in diameter was determined by weighing the membranes before and after immersion in 15 mL of phosphate buffer solution pH 7.4, at 37 °C. The degree of swelling (%) was determined by the ratio of absorbed water mass and dry membrane mass, expressed as a percentage.

### 2.9. Colorimetry

The chromaticity parameters of the biomembranes were measured with a portable colorimeter model CR-30, Minolta Chroma Meter, Minolta Camera Co. Osaka, Japan. Biomembrane samples without and with extracts of *S. adstringens* and *A. cochliacarpa* were analyzed in triplicate at three different points, two peripheral and one central, on a white plate with a CR-A43 calibration standard and a color scale described by the Commission Internationale de L’éclairage (CIE-L*a*b*). The device has been configured to use a D65 light source. The evaluation was made through the parameters L*, a* and b*, where L* represents the luminosity (being L* = 0 black and L* = 100 white) and a* and b*, are the chromatic coordinates (being +a* red, −a* green, +b* yellow, and −b* blue).

### 2.10. Scanning Electron Microscopy (SEM)

The surface morphology and cross-section of the membranes were evaluated by scanning electron microscopy using the FEI Quanta 200 equipment (FEI Company, Amsterdam, The Netherlands) with a vacuum atmosphere of 106 torr. The samples were mounted on aluminum supports with carbon tape, sprayed with a gold film (BALTEC SDC 050, Sputter Coater, Wetzlar, Germany) and observed under a scanning electron microscope. Electromicrographs were generated in topographic mode (secondary electrons) at 9 kV.

### 2.11. Fourier Transform Infrared Absorption Spectroscopy (FTIR)

The FTIR spectra of the membranes were obtained using a Bruker spectrometer, model Vertex 70v, Berlin, Germany, coupled with the surface analysis technique, attenuated total reflection (ATR) that works in diffuse reflectance mode (DRS), in the range of 400 to 4000 cm^−1^, with a resolution of 4.0 cm^−1^.

### 2.12. Thermogravimetric Analysis (TGA)

The thermogravimetry/thermogravimetry derivative (TG/DTG) curves were obtained on a thermobalance (Shimadzu DTG-60, Tokyo, Japan) in the range of 25–500 °C, under a nitrogen atmosphere (N_2_), with a flow rate of gas of 50 mL min^−1^ and heating rate of 10 °C·min^−1^. All analyses were performed using a platinum sample holder containing 2 mg to 2.5 mg of the sample.

### 2.13. In Vivo Healing Biological Assay

#### 2.13.1. Animals and Experimental Groups

A total of 112 male Wistar rats (250 g ± 50 g) were used for the surgical procedure, divided into four groups of 28 animals, placed in cages with shavings bedding, changed daily, kept at a controlled temperature of 22 °C, in a light regime with a light-dark cycle of 12 h, receiving water ad libitum and standard diet Labina^®^ (Purina, São Paulo, Brazil). The animals were subdivided into four groups (*n* = 7): (1) CTR—wounds without coverings; (2) GEL—wounds covered with gelatin membrane; (3) GELSA—wounds covered with gelatin membrane containing hydroacetonic extract of SA) GELAC—wounds covered with gelatin membrane containing hydroacetonic extract of AC.

#### 2.13.2. Surgical Procedure

The wounds were made under general anesthesia, with intraperitoneal injection of 0.1 mL/100 g of a solution composed of 1 mL of ketamine (50 mg) and 1 mL of xylazine (20 mg). The back of the animals was shaved and antisepsis was performed with 1% topical povidone-iodine. Circular dermoepidermal excisions of a dorsal skin flap measuring 8.0 mm in diameter, standardized with a stainless punch scalpel, were performed. All membranes used to cover the wounds measured 1.5 cm in diameter and were fixed to the skin with 0.9% saline. In the immediate postoperative period, the animals received a prophylactic dose of 10 mg/kg of diclofenac potassium intramuscularly. The research complied with the Ethical Principles of Animal Experimentation and was submitted to the Ethics Committee on the Use of Animals (CEUA) of the Tiradentes University, being approved under the number 031114.

#### 2.13.3. Determination of Wound Closure Index

Wounds were photographed with a digital camera (Cybershot Sony HX-300, Tokyo, Japan) fixed on a tripod, standardized height of 20 cm away from the wound, on days 0 (immediately after surgery), 3, 7, 14 days postoperatively. The images were analyzed using the E.A.R Research Project software. The percentage of wound retraction was calculated by the percentage ratio of area decrease in relation to the initial area of the wound.

#### 2.13.4. Removal of Specimens and Histological/Histochemical Procedures

After 3, 7, and 14 days from the surgical procedures, seven animals from each group were euthanized in a CO_2_ chamber (model CGSCO2G—Beiramar). Each wound was dissected with a 1 cm margin of intact skin around the lesion, with depth to the dorsal musculature of the animal, then the specimens were placed in 10% formalin solution (pH 7.4) for 48 h. Subsequently, the pieces were cross-sectioned, dehydrated in alcohol, diaphanized in xylene, embedded in paraffin and then cut into a 5 μm-thick microtome.

#### 2.13.5. Assessment of the Histological Grading of Wound Repair

Formalin-fixed specimens were dehydrated, cleared, and embedded in paraffin, according to routine histological processing techniques. Eighteen serial histological sections (5 µm thick) were obtained from each embedded specimen, three of them (sections 1, 7 and 13) stained in HE (routine staining), and analyzed under conventional optical light, whereas three were stained in Sirius red (sections 4, 10 and 16), and analyzed under polarized light. The histological grading of wound repair was carried out using a semiquantitative scoring scale based on six histological criteria [23], which is presented in Table 1. Ten histological fields from each histological section (200× magnification) were selected and analyzed to obtain the final healing score in each case by adding the scores of the individual criteria. All the histological analysis was performed by two examiners blinded to the groups.

#### 2.13.6. Analysis of Collagen Deposition

For the evaluation of collagen deposition, histological sections stained with Picrosirius were used and analyzed under polarized light under a microscope (OLYMPUS CX 31, Center Valley, PA, USA) with a camera attached. Collagen fibers were analyzed according to their birefringence pattern (greenish/yellow-green characterizing collagen type III and orange, orange-reddish characterizing collagen type I), with the sum of scores up to 4. Morphological appearance was also evaluated (wavy or stretched, thin or thick, short or long) and the architectural arrangement (reticular, parallel or interlaced) also with the sum of scores up to 4. Density was evaluated for the condition of interfibrillar architectural spaces or collagenous tissue with the sum of from scores up to 4.

### 2.14. Statistical Analysis

Data were analyzed for normality using the Shapiro–Wilk test. Differences between means of three or more groups with a Gaussian distribution were analyzed by the test of variance (ANOVA), two-way with Tukey’s post hoc extension. Those with a non-Gaussian distribution were analyzed using the Kruskal–Wallis test, with Dunn’s post hoc extension. A significance level of 5% was used for all analyses, so that the differences observed were considered significant when *p* < 0.05. For the analysis of the biological healing assay, the GraphPad Prism^®^ version 5.0 program was used, and the results were expressed as mean +/− standard error of the mean (s.e.m.).

## 3. Results and Discussion

### 3.1. Extract Characterization

**Figure 1:**
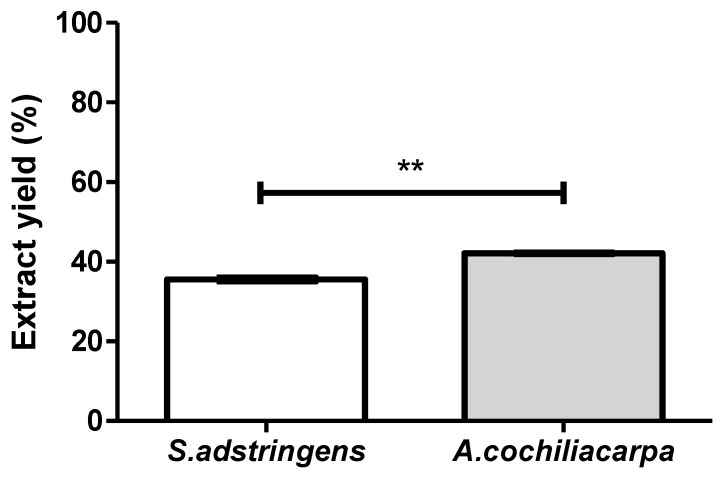
Yields of *S. adstringens* and *A. cochliacarpa* extracts showing a significant difference (** *p* < 0.05) using the *t*-Student test.

The species object of this study, despite being popularly known as “*barbatimão*”, are chemically different, and only *S. adstringens* is recognized as a medicinal plant, allowing the development of products with its active compounds for commercialization. Although both hydroacetonic extracts presented yields above 30%, which is greater than values described in the literature the one obtained from *S. adstringens* was higher than that of *A. cochliacarpa* (Figure 1). The extraction method, as well as the polarity of the extracting solvent, can affect the result. Factors, such as seasonality, temperature conditions, native region of the sample, soil type and material collection period are associated with variations in extract yields [24,25]. Another factor that influences this variation is the exposure to pathogenic agents that can lead to changes in the metabolic pattern of plants, producing phytoalexins or secondary metabolites for their defense and adaptation to environmental conditions [25].

**Figure 2:**
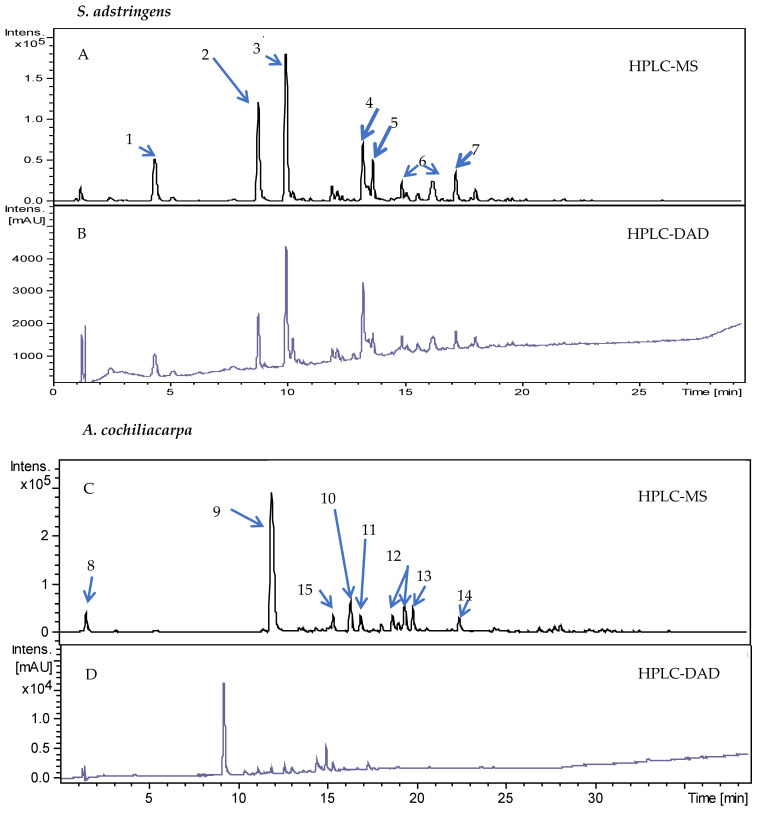
Chromatograms of the hydroacetonic extract of *S. adstringens*—HPLC-MS (**A**) and from HPLC-DAD (**B**); chromatograms of the hydroacetonic extract of *A. cochliacarpa* obtained from HPLC-MS (**C**) and from HPLC-DAD (**D**). Captions: (1) gallocatechin; (2) epigallocatechin; (3) *o*-methyl-epigallocatechin; (4) epigallocatechin; (5) *o*-methyl-epigallocatechin Isomer; (6) di-*o*-ethyl-*prorobinetinidin*-prodelphinidin; (7) *o*-methyl-epigallocatechin-*o*-gallate; (8) di-*o*-hydroxide; (9) catechins and procyanidin *dimers*; (10) *o*-desoxyhexosil-hexoside; (11) procyanidin dimers; (12) procyanidin-*o*-gallate dimers; (13) procyanidin dimers; (14) epigallocatechin; (15) epicatechin.

In the chromatographic analysis, the applied conditions led to a suitable separation of peaks that were identified by mass spectroscopy (Figure 2). It can be observed that the samples of the *S. adstringens* and *A. cochliacarpa* extracts presented polar components. Similar data were found in the study by Lopes et al. [26] with extracts obtained by turbo extraction, the gallocatechin content was 3.8 mg/g, indicating that this method can be applied for the selective determination of polyphenols in extracts of the *S. adstringens* species. The chemical constituents found are also in agreement with those described by Audi et al. [27] for bark and leaves in which gallocatechin, epigallocatechin, 4′-*O*-methyl-gallocatechin and 4′-*O*-methyl-gallocatechin-(4α→8)-4′-*O*-methyl-gallocatechin were identified. Dias et al. [28] found similar results in hydromethanolic fractions of *A. cochliacarpa* extracts in which catechin was isolated at a concentration of 37 mg/g. In another study, Da Silva et al. [29] obtained catechin dimers and trimers and in phytochemical analysis of cold and hot aqueous and methanolic extracts they found saponins, catechins, tannins, phenols, and anthraquinones.

**Figure 3:**
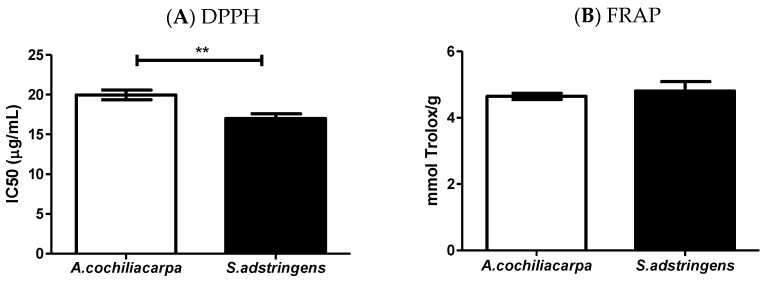
*A. cochliacarpa* and *S. adstringens* extracts obtained by turbo extraction and evaluated by the (**A**) DPPH (** *p* < 0.01) and (**B**) FRAP (*p* > 0.05) methods.

When evaluating the antioxidant activity against the two methods, it is observed that SA presented the best results (Figure 3). This was attributed to the chemical interactions between the solvent used in the preparation of the extract and the presence of substances such as epigallocatechin, gallocatechin, *O*-methyl-epigallocatechin that have suitable electron transfer capacity [30]. In addition to the chemical composition, seasonality and environmental changes can affect the nature of the antioxidant action of certain species [24,25]. Souza et al. [31] studied the potential of antioxidant activity of the *S. adstringens* extract using the DPPH method and observed that the polar extracts showed a high content of total phenols and high antioxidant capacity.

### 3.2. Characterization of Biomembranes

**Figure 4:**
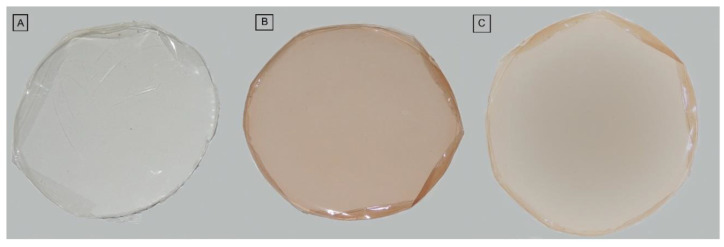
Photographs of gelatin biomembrane—GEL (**A**), gelatin biomembrane containing *S. adstringens* extract—GELSA (**B**) and gelatin biomembrane containing *A. cochliacarpa* extract—GELAC (**C**).

The biomembranes did not present, in macroscopic analysis, insoluble particles. Those containing the *S. adstringens* and *A. cochliacarpa* are translucent and with color homogeneity. The difference in color is due to the compounds present in the extracts (Figure 4).

The analyzed parameters of the main morphological characteristics of the three biomembranes are presented in Table 2. As occurred for the macroscopic properties, the GELAC biomembranes obtained better results than the GELSA and GEL biomembranes, both in handling without breakage and in the ability to bend to breakage. In the evaluation of flexibility and handling, the biomembranes GEL and GELSA presented greater difficulties, being more susceptible to rupture. In contrast, the GELAC biomembrane showed better characteristics, which translates into suitability of the product for everyday use. According to Uriarte-Montoya et al. [32], the balance of the characteristics of polymeric matrices is important for the handling, transport, and storage of wound dressings, without their deformation or rupture, ensuring their protective character. As for the parameters of continuity and homogeneity, the biomembranes were equally excellent according to the score used.

In the determination of the chromatic coordinates “a” and “b” for the analyzed samples, it was observed that there was a difference in the color of the membranes after incorporation of the different extracts (*p* < 0.001), as well as the analysis of luminosity (L) (*p* < 0.0001), as shown in Table 3. The values obtained in GEL were used as a standard to calculate the difference in the “a” and “b” chromaticity coordinates, generating ∆a, which is indicative of the green/red color and ∆b is the blue/yellow color indicator. The same occurred for the calculation of ∆L, which is indicative of luminosity and ∆E, which represents the total difference of the sample color in relation to the GEL.

Analyzing the color difference between the biomembranes suggests that the extracts have different chemical composition since the species are different. The color of the GELSA biomembrane is probably explained by the presence of epigallocatechin, one of its major substances, which has an orange color; Furthermore, according to Drunkler et al. [33], tannic acid and gallic acid are capable of participating in co-pigmentation reactions. Color is also related to the content of total phenols and anthocyanidins. A similar effect was found in the study by Liang et al. [34], when they incorporated esculin (a substance found in the horse chestnut fruit) in a bioactive gelatin film and this increased the difference in color. These authors emphasize that color can influence the final acceptability of the product to consumers. The mechanical and physical properties of the biomembranes are shown in Table 4. Regarding the thickness of the GEL, GELSA, and GELAC biomembranes, significant differences were observed (*p* < 0.05), with GELAC being thicker.

The mechanical properties and permeability indicate that the incorporation of the SA extract reduced the thickness of the GELSA biomembrane, indicating improved material compacting properties. Thickness is an important variable because it influences the uniformity of materials and homogeneity, and is strongly influenced by the casting-type production processes variations, mass/area ratio, and relative humidity of the air and drying [35]. Souza et al. [36] developed bioactive collagen membranes containing red propolis, of similar thicknesses as reported in our study (i.e., 38.9 µm in membranes with collagen only, 22.2 µm for membranes with modified collagen, 28.1 µm for membranes with incorporated extracts).

The ideal thickness depends on the region of the body to be treated [37], and is strongly influenced by the chemical interactions between the extract and the selected plasticizer [38]. Hoque et al. (2011) evaluated the incorporation of cinnamon, clove, and star anise extracts in gelatin-based films and concluded that the increase in film strength is related to the interaction of phenolic compounds present in the extracts with the polymer matrix [39].

Despite the increase in GELSA stiffness, this membrane showed the same deformation profile as the others, indicating that the incorporation of extracts did not influence the membrane extensibility. The degree of extensibility of collagen membranes is strongly dependent on the crosslinking treatments [40]. The chemical composition of the extract and its influence on the molecular structure in which water vapor may or may not flow more easily may also contribute to the low extensibility of GELSA membranes. The water vapor permeability can also be influenced by the plasticizers used in the membrane formulation. When added to other compounds, the extracts can have their physical and mechanical properties altered. The active substances can act alone or together, in the latter case the synergistic effect can often overcome the effects obtained by the isolated active compounds.

**Figure 5:**
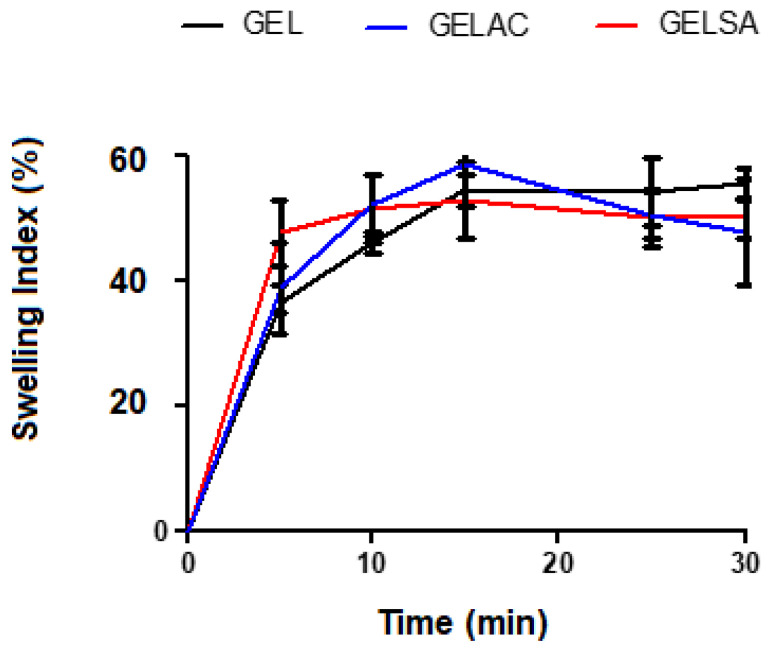
Swelling index (%) of GEL, GELSA, and GELAC biomembranes registered over time up to 30 min.

The swelling curves of the biomembranes are represented in Figure 5. There was no significant difference and all showed water absorption capacity, however, with different absorption intensity. The biomembranes GEL, GELSA, and GELAC started swelling as soon after immersion, with rapid mass gain. The swelling capacity of the biomembranes may have been influenced by the hydrophilic group (OH) found in the polymer matrix, the density of crosslinks, and the presence of pores [41] since there is the possibility of water binding directly to the polymeric chains or filling the spaces in the pores [42,43]. It is also likely that gelatin, interacting with the biomembrane compounds, keeps its molecules further apart, allowing water to enter. It can induce considerable swelling in films due to its solubility and structure formed by a polymeric matrix. This condition is also suggestive of an increase in the active area available in the polymeric mesh, facilitating the absorption of water by hydrophilic chemical groups. Another important factor is the presence of propylene glycol: this organic compound increases both the hydrophilic character and the free volume of the system, favoring increased water absorption. The high degree of swelling observed in biomembranes is due to the presence of gelatin, which has a large number of ionized amino acids in its structure and, consequently, the presence of free charges that favor the entry of water into the polymer matrix [44].

**Figure 6:**
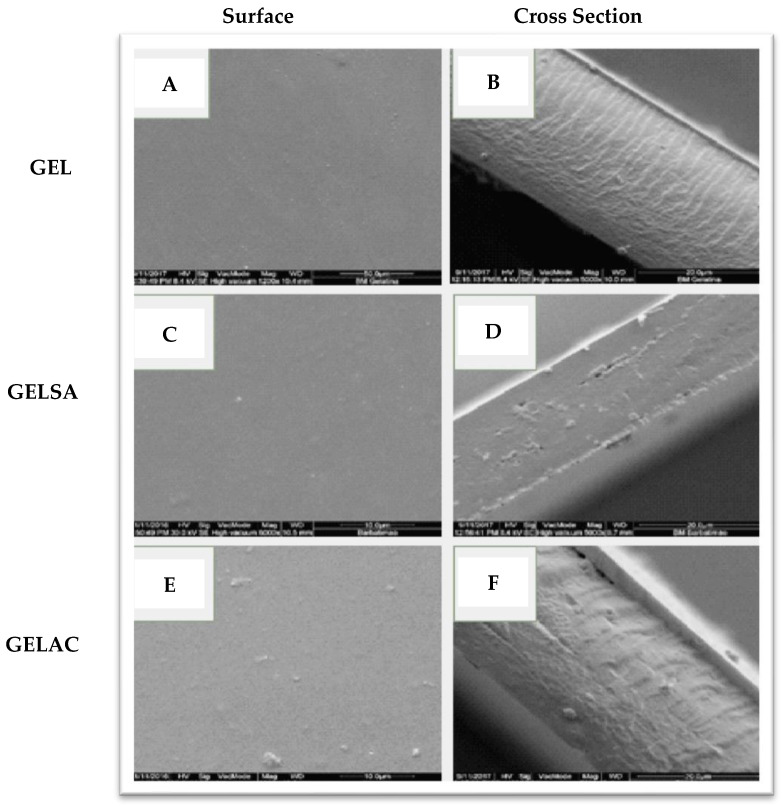
Surface and cross-section micrographs of the GEL (**A**,**B**), GELSA (**C**,**D**), and GELAC (**E**,**F**) biomembranes.

The micrographs of the surfaces and cross-sections of the gelatin biomembranes without and with the addition of extracts are shown in Figure 6. The GEL biomembrane, obtained from gelatin without the addition of extracts, presented a smooth, regular and pore-free surface (Figure 6A, while its cross-section, Figure 6B, presents well-structured and regular channels). In the GELSA biomembrane, the surface presented small pores Figure 6C and in the micrograph of the cross-section, Figure 6D, a compact structure was observed, also with the presence of small pores but maintaining a cohesive structure. In addition, smaller thickness and porosity were observed, indicating the presence of elements that may be interacting with water, such as epigallocatechin. GELAC biomembranes showed a surface with the presence of undissolved substances (Figure 6E), probably the dimers found in the AC extract, which have a higher molecular mass and are possibly less soluble in the aqueous dispersion.

**Figure 7:**
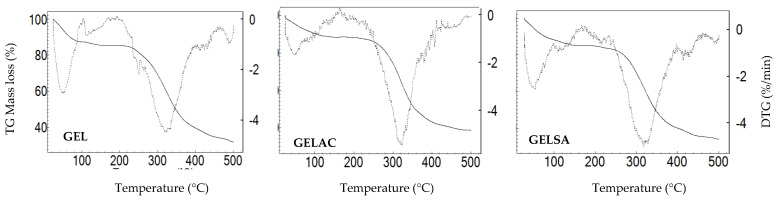
Thermogravimetric (TG, mass loss %) and derivative of thermogravimetric (DTG, %/min) curves of the GEL, GELAC, and GELSA biomembranes, at temperatures up to 500 °C.

In the cross-section of GELAC, a coarser structure was observed, with roughness, random arrangement, and less organization (Figure 6F). This can be attributed to the precipitation of the extract on the surface of the membrane or even the interaction between the chemical structures of catechin with water and propylene glycol. The pores do not fully cross their thickness, a feature that favors the application of the biomaterial in the treatment of skin lesions since the penetration of microorganisms into the wound would be avoided [45,46]. In the cross-sections of GELSA and GELAC, the formation of a denser layer was observed when compared to regular pores formed in GEL. This behavior seems reasonable considering that the chemical structure of the extracts has hydroxyl groups that can be made available for interactions, providing a structural modification in the biomembranes. Indeed, the incorporation of active components in the polymer matrix is influenced by various factors, such as the size and type of the molecule. Rattaya et al. (2009) [47] reported a similar result to this work when they evaluated the incorporation of seaweed extract in gelatin-based films and found that for the additive films, a rougher internal structure and discontinuity zones in relation to films without the addition of the extract, possibly associated with the binding of the phenolic compounds of the extract with the matrix. In the study by Bodini et al. (2013) [48], the incorporation of the ethanolic extract of propolis in gelatin-based films for use as packaging increased the concentration of the extract, which resulted in an increase in the porosity of the matrix, while the film without the addition of the extract formed a more compact and oriented matrix. Thermogravimetric analysis was performed to understand the thermal behavior of biomembranes with extracts of *S. adstringens* and *A. cochliacarpa*. The thermoanalytical curves of the biomembranes of GEL, GELSA, and GELAC are presented in Figure 7. In the TG curve, it is possible to observe that the samples have similar thermal profiles and that for all curves, there is a first event, which characterizes mass loss relative to the loss of moisture and then the onset of degradation. However, between 25 and 100 °C, referring to the dehydration step, there was a greater loss of mass for the GEL sample (12.6%) in relation to GELSA (10.7%) and GELAC (10.5%). This step is related to the loss of surface adsorbed water and water of hydration and can also be attributed to the elimination of proline, present in gelatin. The collagen degradation profile is in agreement with the study by Lakra et al. (2014) [49], where gelatin membranes lost surface water.

The biomembranes show thermal stability without mass loss up to 215 °C. Between 230–380 °C, there is a marked loss of mass that can be attributed to the elimination of amino acid fragments from gelatina [50], which is attributed to the degradation of the structure of biomembranes (GEL, GELSA, and GELAC).

**Figure 8:**
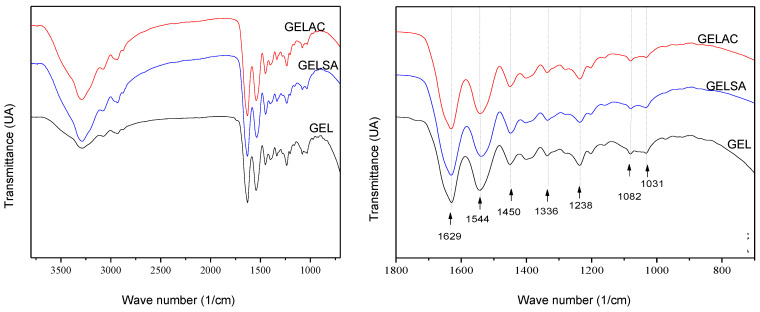
Infrared absorption spectra of GEL, GELAC, and GELSA biomembranes (left-hand side), with detailed analysis of the main peaks (right-hand side).

The FTIR spectra of the GEL, GELSA, and GELAC biomembranes are shown in Figure 8. It was observed that the biomembranes, regardless of whether or not they contain extracts in their constitution, showed typical bands characteristic of the gelatin molecule. Bands at 1629 and 1544 cm^−1^ were observed, which are attributed to the vibrations of amide I (C=O stretching) and amide II, related to the stretching of the C-N group [51]. All membranes showed the same band intensity and none appeared or was suppressed, without significant shifts. The bands at 1544 cm^−1^ can be attributed to free water or type II amides or to an NH bending of the amide II [51,52]. Values between 3150 and 3480 cm^−1^ are characteristic of surface adsorbed water or NH binding present in amino acids/gelatin [51]. The spectra showed a broad band at 3280 cm^−1^ referring to the -OH elongation, a consequence of intramolecular hydrogen bonding [53]. Membranes containing extract showed greater intensity in this band, suggesting greater interaction of intramolecular hydrogen.

A similar result was found in the study conducted by Hoque et al. (2011) [39], which correlated the displacement of amide A from 3275 to 3277 cm^−1^ due to the incorporation of anise extract in gelatin-based films and the interaction of phenolic compounds present in the extract with the polymeric matrix, for them this displacement occurred due to the binding of the phenolic compounds with the NH_2_ groups of the gelatin.

### 3.3. Biological Assay

**Figure 9:**
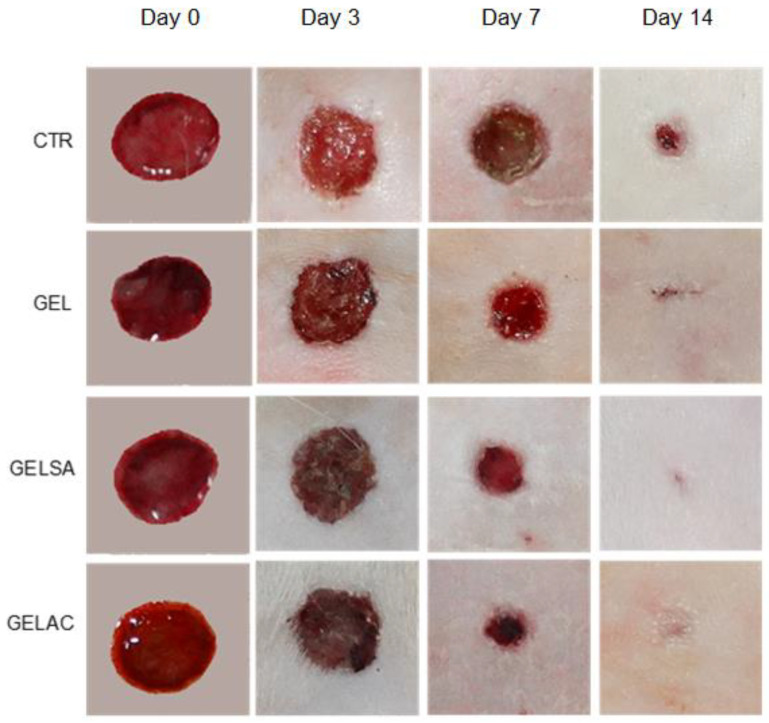
Photographs of the appearance of the dermal wounds during the evaluation of the retraction index of the groups: CTR (control without coverage), GEL (coverage with gelatin membrane), GELSA (gelatin membrane with *S. adstringens* extract), and GELAC (gelatin membrane with *A. cochliacarpa* extract), at 0, 3, 7, and 14 days after wound preparation.

Topical application of biomembranes on dermal lesions in the GEL, GELSA, and GELAC groups and in the CTR group (without coverage) did not promote clinical changes compatible with a deficient repair process, thus, there is no purulent collection in the treated and adjacent areas. It is also visible that the tissue retraction was progressive, being earlier in the groups containing active compounds, over the period of 14 experimental days and, in accordance with the photographic images (Figure 9).

**Figure 10:**
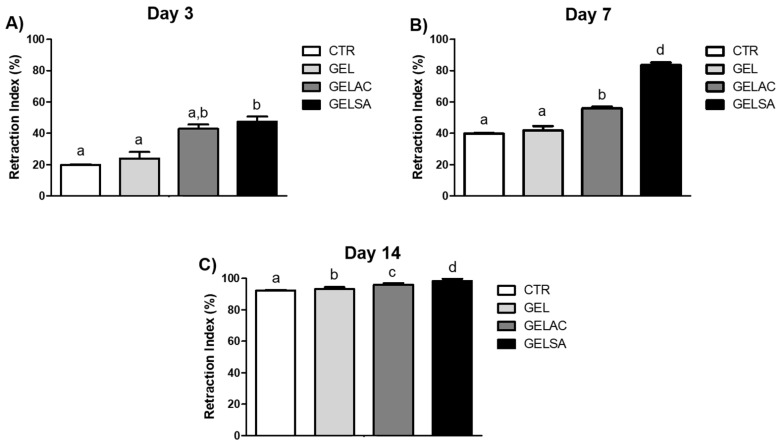
Determination of the average wound retraction indices of the experimental animals on day 3 (**A**), day 7 (**B**), and day 14 (**C**). Data are expressed so that equal letters represent no significant difference and different letters represent a difference of *p* < 0.001.

The average percentage of wound closure on the 3rd (Figure 10A) and 7th (Figure 10A,B) days in the groups treated with GELSA and GELAC biomembranes was significantly higher (*p* < 0.01) when compared to the CTR and GEL groups (*p* < 0.001). In addition, GELAC presented the highest percentage of wound retraction among the treated groups, when compared to CTR, at the two experimental times: around 60% by day 3 and 80% by day 7. At 14 days (Figure 10C), all groups showed a significant difference (*p* < 0.01). In addition, the GELSA and GELAC groups showed the best results, achieving 100% wound retraction.

The use of biomembranes as a therapeutic agent for wounds was based on several previous studies showing that such biomaterials active substances at the site of injury to favor wound repair [10,11,12,21,54].

Biomembrane dressings containing barbatimão extracts accelerated the wound retraction process from the first to the last experimental day, particularly on days three and seven, around 60% and 80%, respectively. As wound closure strongly depends on the progressive reduction in the inflammation and development of the granulation tissue, this process is supposed to be accelerated in wounds treated with anti-inflammatory compounds [55]. In fact, the anti-inflammatory effect of SA was reported by Henriques et al. (2016) [56], who observed a reduction in leukocyte migration and neutrophil accumulation in the knee joint in rats treated with aqueous fractions of the extracts. The anti-inflammatory and antioxidant effect of CA was observed in the study by Sánchez-Fidalgo et al. (2013) [57] when they used the butanolic fraction in rats and concluded that the beneficial effects are probably mediated by the presence of catechins, the majority substance in the extracts. They also observed that the extracts showed a modulatory action on macrophages, promoting less tissue swelling. However, the wound closure improvement observed on day 14 is unlikely related to the direct activity of phytochemicals derived from *S. adstringens* and A. *cochliacarpos* extracts because the biomembranes containing the extracts were fully degraded in approximately 48 h. Therefore, the better rates of wound closure observed in GELSA and GELAC could be an ultimate response to an earlier modulation of the inflammatory phases rather than the result of a possible biological effect on the late stages of wound repair.

**Figure 11:**
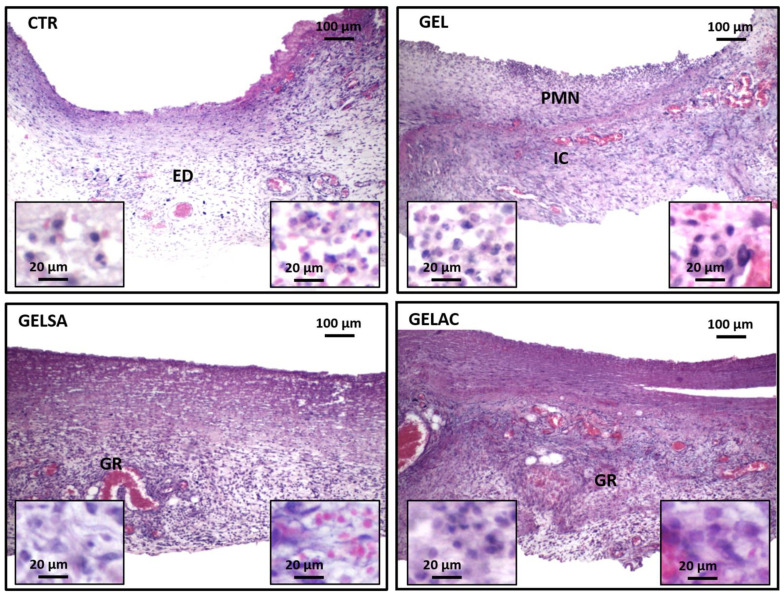
Photomicrographs of HE-stained histological sections representative of the 3-day repair process. CTR shows inflammatory infiltration rich in polymorphonuclear neutrophils associated with intense interstitial edema (100×); highlighted the presence of neutrophil polymorphonuclear leukocytes (small lobulated nuclei) and lymphocytes (dark round nuclei) (400× and 800×). GEL and GELSA present edema and infiltrate of superficial polymorphonuclear neutrophils and a very immature granulation reaction at the base of the wound (100×), while GELAC presents greater fusocellular cellularity of the granulation reaction; highlighted, in the three groups, stromal spindle cells that make up the granulation reaction (400× and 800×). Caption: CTR—control group; GEL—group treated with gelatin biomembranes only; GELSA—group treated with gelatin biomembranes containing *S. adstringens* extract; GELAC—group treated with *A. cochliacarpa* extract; ED—interstitial edema; PMN—polymorphonuclear neutrophils; GR—granulation reaction.

On day 3 (Figure 11), an intense acute inflammatory reaction was observed, with polymorphonuclear neutrophils infiltrating especially the wound surface, in all groups analyzed. At the depth of the lesion, the control group (CTR) presented an intense acute inflammatory reaction, characterized by the presence of edema and serofibrinous exudate. In the GEL, GELSA, and GELAC groups, moderate interstitial edema and marked subacute inflammatory infiltration was observed, composed of polymorphonuclear neutrophils in association with lymphocytes and macrophages. In the GELSA and GELAC groups, there was formation of immature granulation reaction, rich in hyperemic capillaries at the base of the wound at the same time interval. However, the GELAC group stood out for the greater amount of stromal cells distributed throughout the wound area.

**Figure 12:**
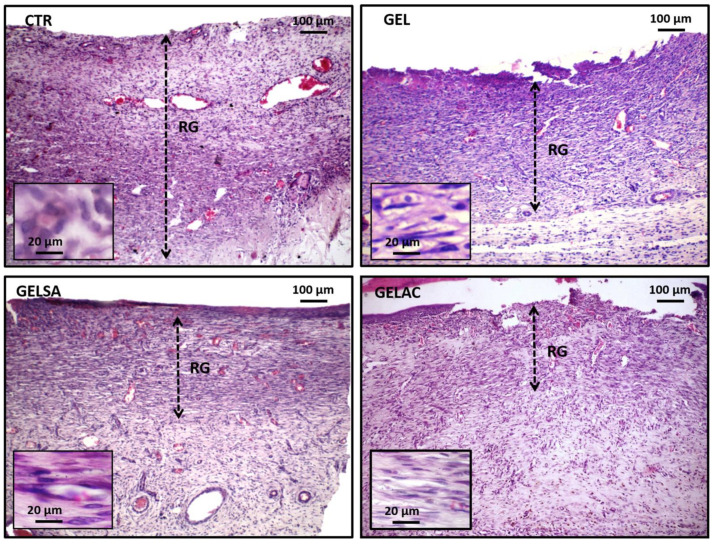
Photomicrographs of HE-stained histological sections representative of the 7-day repair process. All groups present exuberant granulation reactions occupying the repair area. CTR exhibits a granulation reaction band that extends from the hypodermis to the wound surface (“top to bottom”), whereas in GEL, it occupies approximately 2/3 superficial depth of the injured area (100×). In GELSA and GELAC, the granulation reaction range occupies approximately 1/3 of the depth extension of the injured area (100×). Highlighted detail of the cells of the granulation reaction, consisting of fusiform and inflammatory cells in CTR and GEL, but essentially fusocellular in GELSA and GELAC (400×). Caption: CTR—control group; GEL—group treated with gelatin biomembranes only; GELSA—group treated with gelatin biomembranes containing *S. adstringens* extract; GELAC—group treated with *A. cochliacarpa* extract; GR—granulation reaction; dotted arrow—thickness, in depth, of the granulation reaction band.

On day 7 (Figure 12), all groups presented an area of exuberant granulation reaction with predominantly chronic inflammatory infiltrate, in which a high number of small irregular blood vessels perpendicular to the wound surface was evidenced. The intense proliferation of spindle cells was also observed, interpreted as young fibroblasts, myofibroblasts, and endothelial cells, organized in regular series, distributed along thin eosinophilic collagen fibrils arranged parallel to the wound surface. The CTR group presented the most extensive range of granulation reactions corresponding to approximately 2/3 of the total area of the lesion, while in the deepest 1/3, there was greater collagen deposition at the expense of inflammatory infiltration and neovascularization. GEL exhibited a thick granulation reaction range, although smaller than the CTR, with the presence of well dilated and congested capillaries, the presence of greater collagen tissue density, and a reduction in the intensity of the inflammatory process, which suggests a more mature granulation reaction. The GELSA and GELAC groups showed a significant reduction in the thickness of the granulation reaction band, greater fusocellular and collagen condensation, and lower vascular contingent when compared to the CTR and GEL groups.

**Figure 13:**
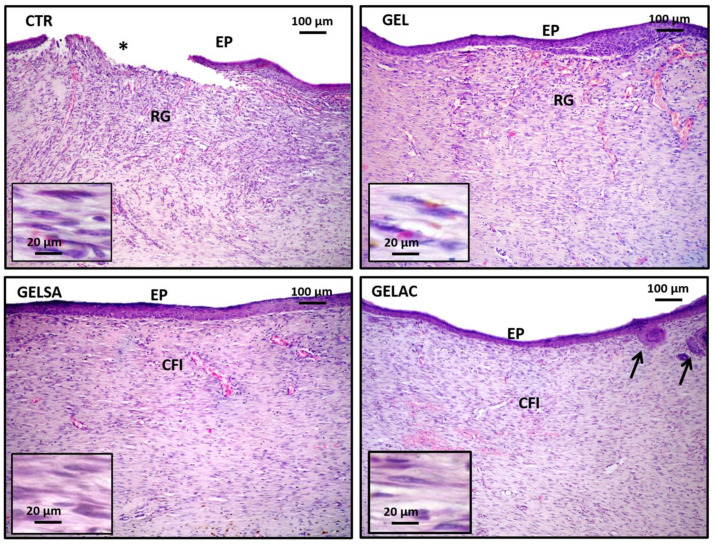
Photomicrographs of HE-stained histological sections representative of the 14-day repair process. CTR shows a residual granulation reaction arranged in a still well-vascularized surface band and areas of incomplete epithelialization (100×). (400×). GEL exhibits residual granulation reaction also richly vascularized, but with complete epithelialization (100×). GELSA and GELAC present an immature, hypovascularized, and richly cellular fibrous scar (100×). Notes formation of marginal epithelial buddings compatible with rudimentary skin appendages in GELAC (arrows). Highlights show details of the spindle cells and capillaries composing the residual granulation reaction in CTR and GEL and of the predominant spindle cell component in the immature fibrous scar in GELSA and GELAC (400×). Caption: CTR—control group; GEL—group treated with gelatin biomembranes only; GELSA—group treated with gelatin biomembranes containing *S. adstringens* extract; GELAC—group treated with *A. cochliacarpa* extract; GR—granulation reaction; PE—keratinized stratified squamous epithelium (epithelialization); IFC—immature fibrous scar; (*)—area of denudation of connective tissue resulting from incomplete epithelialization.

On day 14 (Figure 13), the CTR group exhibited a granulation reaction with a thick band in about 1/3 of the wound depth, richly cellular and hypovascular. Although all cases showed advanced epithelialization, in two animals, it was incomplete. The GEL and GELSA groups exhibited a very thin subepithelial band of granulation reaction, with small residual capillaries present, while the entire remaining area of the wound was constituted by a well-collagenized fibrous scar with fibroblastic cells and a hypovascularized area. GELAC, in turn, presented a well-collagenized fibrous scar, very thin, and dense throughout the wound area, with a relatively homogeneous distribution of fusiform and ovoid cells, identified as fibroblasts. In all groups treated with gelatin coating, regardless of the incorporation of actives (GEL, GELSA, and GELAC), epithelialization was complete; however, in GELAC, small marginal epithelial buddings (buddings) were evidenced, which were interpreted as the formation of rudiments of skin appendages, such as hair follicles.

Wound repair is characterized by a dynamic process that involves the phases of epithelialization, proliferation, and fibroplasty [58,59]. On the third day, an intense inflammatory reaction is expected since soon after the injury, a response to vascular and biochemical changes occurs, stimulating the arrival of neutrophils in the injured area with the purpose of preventing invasion and proliferation [59]. These data, therefore, justify the inflammatory infiltration predominantly rich in polymorphonuclear neutrophils, edema, and hyperemia observed in the present study. The presence of granulation reaction in the depth of the wounds observed in the GELSA and GELAC groups suggests an acceleration in the repair, which corroborates the clinical data of wound closure previously presented in this study. Therefore, it is possible to suggest that the leukocyte migration and neutrophil accumulation in injured tissues were inhibited by *S* from *S. adstringens* and A. *cochliacarpa* extracts [56,57], favoring the earlier development of the granulation tissue. In addition, polymorphonuclear neutrophils, the main leukocyte cells involved in the acute inflammatory response, are strongly associated with the induction of oxidative stress in conditions of tissue injury [60,61]. Thus, the best histopathological features seen in the GELSA and GELAC groups in the earlier stages of repair could be a result of the lower intensity of inflammation and oxidative stress in the wound area.

**Figure 14:**
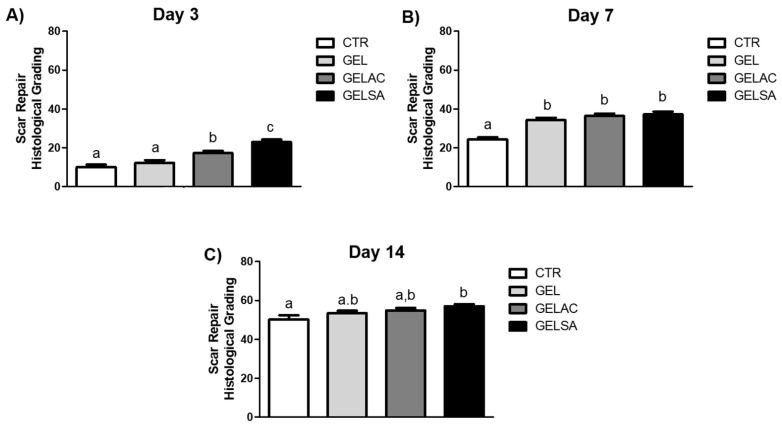
Mean scores of the evolutionary profile of the wound repair process in rats submitted to treatment with biomembranes containing SA extract and AC extract, on (**A**) day 3, (**B**) day 7 and (**C**) day 14. Data expressed as mean ± standard error of the mean. Equal letters represent no significant difference (*p* > 0.05), different letters represent significant difference *p* < 0.001.

The mean histological grading scores of the surgical wound repair process of the CTR, GEL, GELSA, and GELAC groups at 3, 7, and 14 days are described and represented below, in accordance with Figure 14. The overall beneficial effect of the GELSA and GELAC treatment on the histological features of wound repair was well demonstrated by the histological grading of wound repair. The scoring system used in the current work was designed in such a way that lower scores are associated with unhealed wounds, whereas higher scores are associated with completely closed and healed wounds without excessive scarring [23]. GELSA and GELAC presented higher scores of histological grading, suggestive of wound repair improvement due to reduced inflammation, earlier maturation of the granulation tissue, and better epithelization and scarring. These data suggest that the application of the biomembranes GELSA and GELAC not only accelerated the clinical wound closure but also improved the histological features of wound healing.

**Figure 15:**
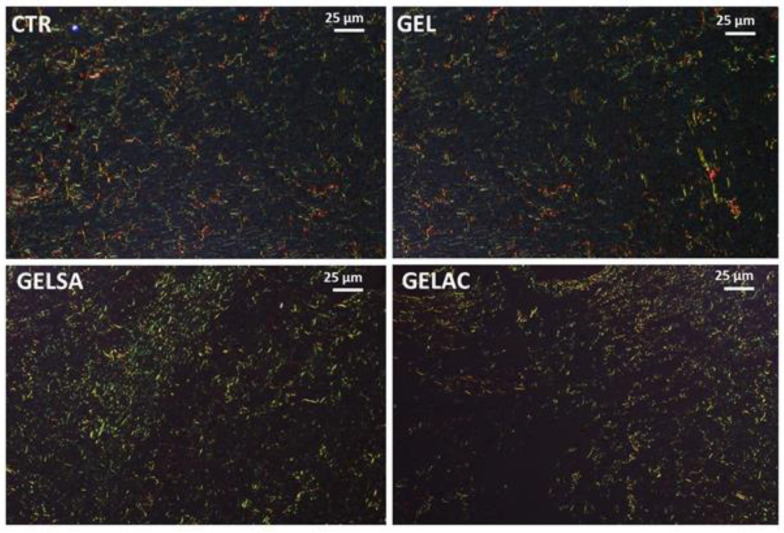
Photomicrographs of histological sections stained in picrosirius and analyzed under polarized light representative of the 3-day repair process. All groups present thin and short collagen fibrils with greenish-yellow birefringence compatible with type III collagen, arranged in a reticular pattern; the interfibrillar spaces (dark) were large and abundant (400×). Caption: CTR—control group; GEL—group treated only with gelatin biomembranes; GELSA—group treated with gelatin biomembranes containing *S. adstringens* extract; GELAC—group treated with *A. cochliacarpa* extract.

On the third day, Picrosirius Red-stained sections examined under polarized light revealed, in all groups, the presence of rare, predominantly short, thin, and delicate fibrous bundles with greenish birefringence suggestive of type III collagen throughout the wound area. These fibrils seemed to be arranged in a reticular organization, resembling a “spider’s web” arrangement. The interfibrillar spaces were abundant, wide, and irregular (Figure 15). No apparent difference was observed between the groups.

**Figure 16:**
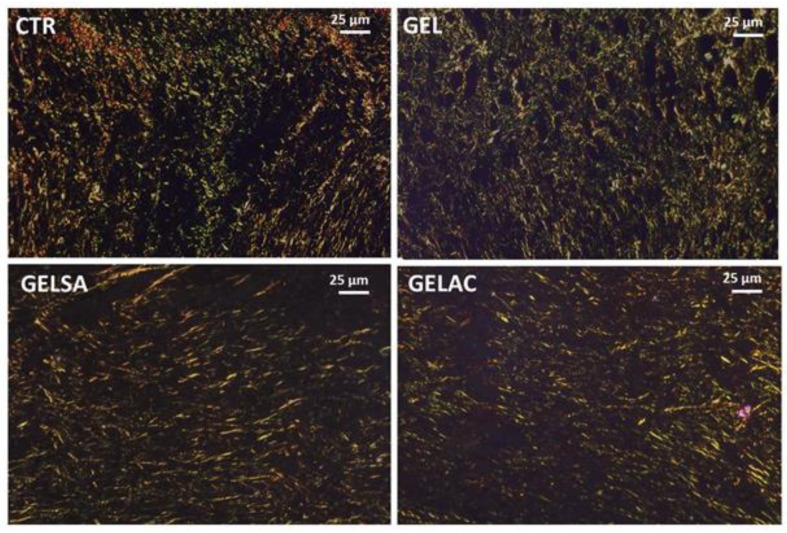
Photomicrographs of histological sections stained in picrosirius and analyzed under polarized light representative of the 7-day repair process. All groups present thin and short collagen fibrils with yellow-green birefringence compatible with type III collagen, interspersed with thicker fibers with golden birefringence, consistent with type I collagen, arranged in a predominantly reticular pattern in CTR and GEL, but with areas parallel (especially in wound depth) in GELSA and GELAC; the interfibrillar spaces (dark) were notably smaller but still abundant (400×). Caption: CTR—control group; GEL—group treated only with gelatin biomembranes; GELSA—group treated with gelatin biomembranes containing *S. adstringens* extract; GELAC—group treated with *A. cochliacarpa* extract.

On day 7, all groups presented a very delicate weave of short, thin, and delicate collagen fibrils showing a dual birefringence pattern, comprising molecules with a more greenish glow, compatible with type III collagen, permeated by others with a golden-yellow glow, consistent with type I collagen. The interfibrillar spaces were still wide, although more regular, in the CTR and GEL groups. In the GELSA and GELAC groups, on the other hand, the collagen network was more compact, being formed by crisscrossed birefringent fibrils giving a “meshed” appearance, with smaller interfibrillar spaces between them (Figure 16).

**Figure 17:**
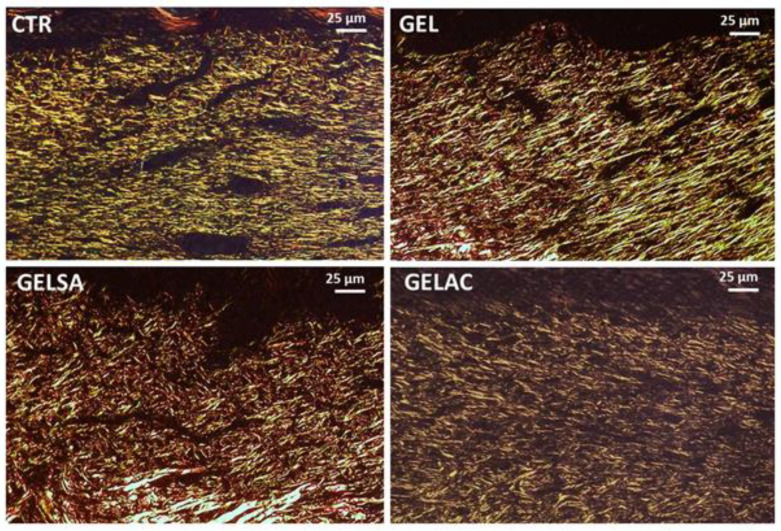
Photomicrographs of histological sections stained in picrosirius and analyzed under polarized light representative of the 14-day repair process. All groups present thicker collagen fibers of variable length with golden and reddish birefringence, consistent with type I collagen, arranged in a pattern predominantly parallel to the wound surface; the interfibrillar spaces (dark) were very reduced (400×). Caption: CTR—control group; GEL—group treated only with gelatin biomembranes; GELSA—group treated with gelatin biomembranes containing *S. adstringens* extract; GELAC—group treated with *A. cochliacarpa* extract.

On the 14th day (Figure 17), all groups presented collagen fibers with predominantly yellow-orange and reddish birefringence compatible with type I collagen, which were organized in a reticular architectural arrangement on the surface of the wounds, but notably parallel in depth. In the GEL, GELSA, and GELAC groups, the fibers are seen to be densely arranged, intertwined, compacted, and with reduced interfibrillar spaces, characterizing a greater density and collagenization maturity.

**Figure 18:**
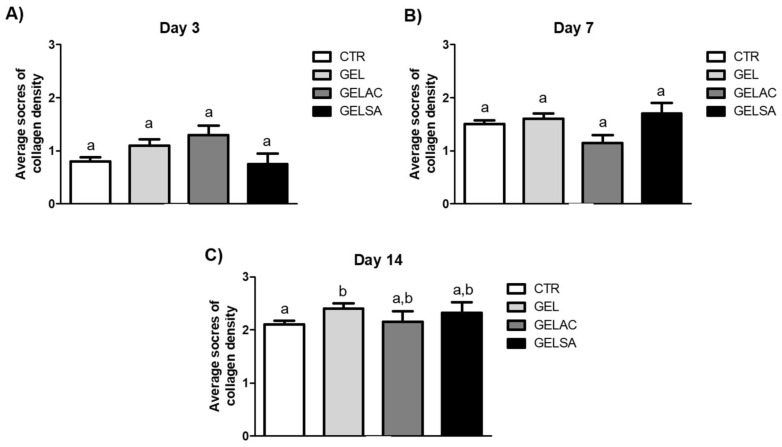
Mean scores of collagen density arranged in histological sections of the healing process of open wounds at (**A**) 3, (**B**) 7, and (**C**) 14 days (Sirius Red/polarized light, 400× magnification). Equal letters represent no significant difference. Different letters represent a significant difference of *p* < 0.05 (one-way ANOVA test followed by Tukey’s test).

Figure 18 shows the mean collagen density scores of the surgical wound repair process in the CTR, GEL, GELSA, and GELAC groups at 3, 7, and 14 days. At 3 and 7 days, there was no significant difference between the groups. At 14 days, only the GEL group showed a difference (*p* < 0.05) when compared to the (CTR) group.

Epicatechins have also been shown to accelerate the phenomenon of angiogenesis and fibrous scar formation in experimental wound healing trials in rodents, probably in response to the increased release of vascular-endothelial growth factor (VEGF) [62,63]. In addition, these polyphenols also induce an increase in the release of TGF-β, a cytokine closely related to the differentiation of fibroblasts into myofibroblasts and the synthesis and deposition of scar collagen [64]. From another perspective, a previous study carried out in a wound healing model in rodents showed that the use of methanolic extract of *Camellia sinensis*, rich in catechins and flavonoids, promoted better wound retraction rates and histological evidence of acceleration of repair steps, such as formation and maturation of the granulation reaction and scar collagen deposition [65]. Furthermore, catechins isolated from *Parapiptadenia rigida* showed promising results regarding the stimulation of fibroblast proliferation and migration in in vitro assays (scratch assay), suggesting potential application as a healing-accelerating agent [66]. These reports seem to support the histological findings observed in the present study since the use of biomembranes containing extracts rich in epicatechins (GELSA) and catechins (GELAC) promoted acceleration in several stages of the repair process over 7 and 14 days. However, further investigations are still required to determine whether the suitable performance of the SA and CA-containing biomembranes can be exclusively attributed to the anti-inflammatory/antioxidant effects of the extracts or whether the biomaterials could act in other stages of wound repair, such as the formation of granulation tissue and fibroblast/myofibroblasts differentiation.

It should be noted that the findings obtained in GELAC were more expressive than in GELSA. However, whether these data are the result of a better healing potential of catechins in relation to epicatechins, or if they represent the action of other chemical compounds present in each type of extract, still needs to be investigated in further studies.

## 4. Conclusions

The data obtained in the present study suggest that the use of GELSA and GELAC biomembranes is able to accelerate the healing process, especially in the early (three days) and late (14 days) phases. These data corroborate those observed in the descriptive histological analysis and reinforce the possible involvement of the active compounds present in the extracts in the stimulation of the healing stages. Furthermore, GELAC showed the best grading results at the end of the experimental period, which was also apparent in the previous descriptive analysis. The histological healing stages that occur around the 14th day of repair in a rodent model are represented by the phenomena of fibroblast proliferation and collagen deposition [67]. Therefore, it would be possible to hypothesize that catechins, the major compounds present in GELAC, could play a role in fibroproliferative healing dynamics.

However, it is necessary to consider that the degradation of biomembranes occurs in the first 48 h of application. Thus, the possible effects must be concentrated in the initial phases of the repair since, in the final stages, there is no longer any presence of actives at the dermal injury site. In this sense, Tanigawa et al. (2014) [68] demonstrated that catechins are able to protect fibroblasts (NIH3T3 strain) from apoptosis induced by oxidative stress caused by reactive oxygen species. Silva Santos et al. (2017) [69] reported that catechins reduce mitochondrial dysfunction and amiodarone-induced cellular oxidative stress in lung fibroblasts. Additionally, Morin and Grenier (2017) [70] demonstrated that treatment with catechin-rich green tea extract (*Camellia sinensis*) in the coculture of gingival fibroblasts and macrophages stimulated by *A. actinomycetemcomitans* reduced the synthesis of metalloproteinases (MMP-3 and MMP- 9). These data available in the literature lead to the hypothesis that the best results obtained with GELAC in 14 days could be a reflection not of the direct action of catechins on fibroplasia at the end of the experimental period but of a possible protective effect on these cells against stress. Oxidative effect in the initial periods, facilitating its later proliferation, as well as the modulation of collagen degradation induced by metalloproteinases. This hypothesis also finds support in the fact that the main clinical and morphological findings were evidenced in the first repair phase (3 days), so the subsequent steps would have been accelerated by the lower intensity of the inflammatory reaction and consequent minimization of oxidative stress and its consequences effects on proliferating fibroblasts. Taken together, the results of the present study suggest that the biomembrane with extracts of *S. adstringens* and *A. cochliacarpa* may represent promising therapeutic strategies for the treatment of wounds in which there was no possibility of edge coaptation. The data obtained still seem to point to a better result from the use of biomembranes containing *A. cochliacarpa*. Although some mechanisms have been hypothesized to explain the nature of the results, further studies are still needed in order to elucidate the possible pathophysiological processes behind the acceleration of wound repair. The results show that there was an improvement in healing and that the compounds released at the beginning of the phase stimulated the healing process throughout the entire period. Macroscopically, it was observed that the biomembranes were degraded in 48 h. The reasons that determine this increase in wound repair rates are still unclear. However, it is suggested that the compounds present in the biomembranes contributed significantly to wound closure. These data are a strong indication that biomembranes with barbatimão compounds actually have an important clinical effect on the repair process. Biomembranes had a positive effect both histologically and on the repair process that warrants further study. The effect of the use of biomembranes also occurs in terms of the quality of the scar formed. This gives rise to the need to study how this scar is organized, for which a histological analysis was performed. It is suggested that the polyphenols identified in this study, such as catechin and epigallocatechin, favor the reduction in pro-inflammatory proteins and accelerate the healing process.

## Figures and Tables

**Table 1:** Parameters assessed to calculate wound repair score.

Histological Criteria	Score System	Histological Method
Inflammatory infiltrate	Plenty—1, moderate—2, a few—3	Light microscopy (HE)
Amount of granulation tissue	Profound—1, moderate—2, scanty—3, absent—4	Light microscopy (HE)
Orientation of collagen fibers	Vertical—1, mixed—2, horizontal—3	Polarized light (sirius red)
Pattern of collagenization	Reticular—1, mixed—2, fascicle—3	Polarized light (sirius red)
Amount of early collagen (type III)	Profound—1, moderate—2, minimum—3, absent—4	Polarized light (sirius red)
Amount of mature collagen (type I)	Profound—1, moderate—2, minimum—3	Polarized light (sirius red)

**Table 2:** Main morphological characteristics observed in GEL, GELSA, and GELAC biomembranes.

Biomembranes	Continuity	Homogeneicity	Handiness	Flexibility
GEL	+++	+++	++	++
GELSA	+++	+++	++	++
GELAC	+++	+++	+++	+++

(++) good, (+++) excellent. GEL: gelatin biomembrane; GELSA: biomembrane with *S. adstringens* extract; GELAC: biomembrane with *A. cochliacarpa* extract.

**Table 3:** Mean values of the color parameters obtained in the colorimetric analysis on a white background of the GEL, GELSA, and GELAC biomembranes. Different letters in the same column represent statistically different values. *p* < 0.001.

Membrane	∆L	∆a	∆b	∆E
GEL	0	0	0	0
GELSA	13.85 ± 0.94 ^(a)^	−7.48 ± 0.61 ^(a)^	−14.73 ± 0.95 ^(a)^	21.56 ± 1.41 ^(a)^
GELAC	8.42 ± 1.46 ^(b)^	−4.53 ± 0.99 ^(b)^	−11.59 ± 1.57 ^(b)^	15.04 ± 2.24 ^(b)^

**Table 4:** Properties of GEL, GELSA, and GELAC biomembranes. Different letters on the same line represent significantly different values (*p* < 0.05).

Parameter	GEL	GELSA	GELAC
Thickness (µm)	27.41 ± 0.4 (a)	17.43 ± 0.18 (b)	29.94 ± 0.38 (c)
Young’s Modulus (MPa)	1185.06 ± 268.7 (a)	2387.74 ± 321.09 (b)	1084.95 ± 248.35 (a)
Maximum Voltage (MPa)	43.33 ± 9.0 (a)	65.32 ± 20.76 (b)	32.28 ± 15.11 (a)
Deformation (%)	7.12 ± 2.3 (a)	6.2 ± 1.99 (a)	5.4 ± 1.84 (a)
Permeability (g·mm/day·m^2^·KPa)	9.68 ± 3.2 (a)	10.39 ± 2.77 (a)	11.82 ± 1.83 (b)

## Data Availability

Not applicable.

## References

[1] Sen C.K. (2019). Human Wounds and Its Burden: An Updated Compendium of Estimates. Adv. Wound Care.

[2] Laurano R., Boffito M., Ciardelli G., Chiono V. (2022). Wound dressing products: A translational investigation from the bench to the market. Eng. Regen..

[3] Rezvani Ghomi E., Khalili S., Nouri Khorasani S., Esmaeely Neisiany R., Ramakrishna S. (2019). Wound dressings: Current advances and future directions. J. Appl. Polym. Sci..

[4] Zou F., Sun X., Wang X. (2019). Elastic, hydrophilic and biodegradable poly (1, 8-octanediol-co-citric acid)/polylactic acid nanofibrous membranes for potential wound dressing applications. Polym. Degrad. Stab..

[5] Ndlovu S.P., Ngece K., Alven S., Aderibigbe B.A. (2021). Gelatin-Based Hybrid Scaffolds: Promising Wound Dressings. Polymers.

[6] Tottoli E.M., Dorati R., Genta I., Chiesa E., Pisani S., Conti B. (2020). Skin Wound Healing Process and New Emerging Technologies for Skin Wound Care and Regeneration. Pharmaceutics.

[7] Jaul E., Barron J., Rosenzweig J.P., Menczel J. (2018). An overview of co-morbidities and the development of pressure ulcers among older adults. BMC Geriatr..

[8] Nunes P.S., Albuquerque R.L., Cavalcante D.R., Dantas M.D., Cardoso J.C., Bezerra M.S., Souza J.C., Serafini M.R., Quitans L.J., Bonjardim L.R. (2011). Collagen-based films containing liposome-loaded usnic acid as dressing for dermal burn healing. J. Biomed. Biotechnol..

[9] Nunes P.S., Rabelo A.S., Souza J.C., Santana B.V., da Silva T.M., Serafini M.R., Dos Passos Menezes P., Dos Santos Lima B., Cardoso J.C., Alves J.C. (2016). Gelatin-based membrane containing usnic acid-loaded liposome improves dermal burn healing in a porcine model. Int. J. Pharm..

[10] Santos T.S., Santos I., Pereira-Filho R.N., Gomes S.V.F., Lima-Verde I.B., Marques M.N., Cardoso J.C., Severino P., Souto E.B., Albuquerque-Junior R.L.C. (2021). Histological Evidence of Wound Healing Improvement in Rats Treated with Oral Administration of Hydroalcoholic Extract of Vitis labrusca. Curr. Issues Mol. Biol..

[11] Yoshida C.M.P., Pacheco M.S., de Moraes M.A., Lopes P.S., Severino P., Souto E.B., da Silva C.F. (2021). Effect of Chitosan and Aloe Vera Extract Concentrations on the Physicochemical Properties of Chitosan Biofilms. Polymers.

[12] do Nascimento M.F., Cardoso J.C., Santos T.S., Tavares L.A., Pashirova T.N., Severino P., Souto E.B., Albuquerque-Junior R.L.C. (2020). Development and Characterization of Biointeractive Gelatin Wound Dressing Based on Extract of Punica granatum Linn. Pharmaceutics.

[13] de Carvalho F.M.d.A., Schneider J.K., de Jesus C.V.F., de Andrade L.N., Amaral R.G., David J.M., Krause L.C., Severino P., Soares C.M.F., Caramão Bastos E. (2020). Brazilian Red Propolis: Extracts Production, Physicochemical Characterization, and Cytotoxicity Profile for Antitumor Activity. Biomolecules.

[14] Loureiro K.C., Barbosa T.C., Nery M., Chaud M.V., da Silva C.F., Andrade L.N., Correa C.B., Jaguer A., Padilha F.F., Cardoso J.C. (2020). Antibacterial activity of chitosan/collagen membranes containing red propolis extract. Pharmazie.

[15] Bene K., Sinan K.I., Zengin G., Diuzheva A., Jekő J., Cziáky Z., Aumeeruddy M.Z., Xiao J., Mahomoodally M.F. (2019). A multidirectional investigation of stem bark extracts of four African plants: HPLC-MS/MS profiling and biological potentials. J. Pharm. Biomed. Anal..

[16] Nik Salleh N.N.H., Othman F.A., Kamarudin N.A., Tan S.C. (2020). The Biological Activities and Therapeutic Potentials of Baicalein Extracted from Oroxylum indicum: A Systematic Review. Molecules.

[17] Shamala T., Surendra B.S., Chethana M.V., Bolakatti G., Shanmukhappa S. (2022). Extraction and isolation of Isoflavonoids from stem bark of *Bauhinia purpurea* (L): Its biological antipsychotic and analgesic activities. Smart Mater. Med..

[18] Oliveira R.F., Ribeiro P.R., Santos G.K.M., Oliveira C.S., Silva P.R.C., Oliveira H.A., Trindade R.d.C., Fernandez L.G. (2013). Evaluation of the hepatotoxicity of Abarema cochliacarpos extracts in mice Mus musculus. Rev. Bras. De Farmacogn..

[19] Pellenz N.L., Barbisan F., Azzolin V.F., Santos Marques L.P., Mastella M.H., Teixeira C.F., Ribeiro E.E., da Cruz I.B.M. (2019). Healing activity of Stryphnodendron adstringens (Mart.), a Brazilian tannin-rich species: A review of the literature and a case series. Wound Med..

[20] Nascimento C.A., Santos A.C.M.d., Silva D.M.d., Barbosa N.R., Moura E.L.d., Balliano T.L., Figueiredo E.V.M.d.S., Farias K.F.d., Pitta G.B.B. (2021). Evidence about properties of the extract of Stryphnodendron adstringens (Mart.) Coville (Barbatimão) for clinical practice. Res. Soc. Dev..

[21] Debone H.S., Lopes P.S., Severino P., Yoshida C.M.P., Souto E.B., da Silva C.F. (2019). Chitosan/Copaiba oleoresin films for would dressing application. Int. J. Pharm..

[22] Brand-Williams W., Cuvelier M.E., Berset C. (1995). Use of a free radical method to evaluate antioxidant activity. LWT-Food Sci. Technol..

[23] Gupta A., Kumar P. (2015). Assessment of the histological state of the healing wound. Plast. Aesthetic Res..

[24] Lopes G.C., Sanches A.C.C., Nakamura C.V., Dias Filho B.P., Hernandes L., Mello J.C.P.d. (2005). Influence of extracts of Stryphnodendron polyphyllum Mart. and Stryphnodendron obovatum Benth. on the cicatrisation of cutaneous wounds in rats. J. Ethnopharmacol..

[25] Bisbis M.B., Gruda N., Blanke M. (2018). Potential impacts of climate change on vegetable production and product quality—A review. J. Clean. Prod..

[26] Lopes G.C., Vieira Machado F.A., Mendes de Toledo C.E., Sakuragui C.M., Palazzo de Mello J.C. (2008). Chemotaxonomic significance of 5-deoxyproanthocyanidins in Stryphnodendron species. Biochem. Syst. Ecol..

[27] Audi E.A., Mendes De Toledo C.E., Solera Dos Santos F., Bellanda P.R., Alves-Do-Prado W., Ueda-Nakamura T., Nakamura C.V., Sakuragui C.M., Bersani-Amado C.A., Palazzo De Mello J.C. (2004). Biological activity and quality control of extract and stem bark from Stryphnodendron adstringens. Acta Farm. Bonaer..

[28] Dias A.S., Lima A.C.B., Santos A.L.M.L., Rabelo T.K., Serafini M.R., Andrade C.R., Fernandes X.A., Moreira J.C.F., Gelain D.P., Estevam C.S. (2013). Redox properties of Abarema cochliacarpos (Gomes) Barneby & Grime (Fabaceae) stem bark ethanol extract and fractions. Nat. Prod. Res..

[29] da Silva M.S., Sánchez-Fidalgo S., Talero E., Cárdeno A., da Silva M.A., Villegas W., Souza Brito A.R., de La Lastra C.A. (2010). Anti-inflammatory intestinal activity of Abarema cochliacarpos (Gomes) Barneby & Grimes in TNBS colitis model. J. Ethnopharmacol..

[30] Skrzydlewska E., Ostrowska J., Farbiszewski R., Michalak K. (2002). Protective effect of green tea against lipid peroxidation in the rat liver, blood serum and the brain. Phytomedicine Int. J. Phytother. Phytopharm..

[31] Souza T.M., Severi J.A., Silva V.Y.A., Santos E., Pietro R.C.L.R. (2009). Bioprospecção de atividade antioxidante e antimicrobiana da casca de Stryphnodendron adstringens (Mart.) Coville (Leguminosae-Mimosoidae). Rev. Ciências Farm. Básica E Apl..

[32] Uriarte-Montoya M.H., Arias-Moscoso J.L., Plascencia-Jatomea M., Santacruz-Ortega H., Rouzaud-Sández O., Cardenas-Lopez J.L., Marquez-Rios E., Ezquerra-Brauer J.M. (2010). Jumbo squid (*Dosidicus gigas*) mantle collagen: Extraction, characterization, and potential application in the preparation of chitosan-collagen biofilms. Bioresour. Technol..

[33] Drunkler D.A., Fallcão L.D., Bordignon-Luiz M.T. (2004). Influence of the tannic and gallic acids on stability of betacyanins from red beetroot (*Beta vulgaris* L.) crude extract. Alim Nutr..

[34] Liang C., Ju W., Pei S., Tang Y., Xiao Y. (2017). Pharmacological Activities and Synthesis of Esculetin and Its Derivatives: A Mini-Review. Molecules.

[35] Wang J., Song H., Ren L., Talukder M.E., Chen S., Shao J. (2022). Study on the Preparation of Cellulose Acetate Separation Membrane and New Adjusting Method of Pore Size. Membranes.

[36] Souza I.C.L., Do Nascimento M.F., De Souza Neta R.G., Dos Santos J.E.C., Costa L.P., Cardoso J.C., De Albuquerque-Junior R.L.C. (2013). Effect of the maltodextrin-induced chemical reticulation on the physical properties and healing potential of collagen-based membranes containing Brazilian red propolis extract. Int. J. Med. Med. Sci..

[37] Walles T., Herden T., Haverich A., Mertsching H. (2003). Influence of scaffold thickness and scaffold composition on bioartificial graft survival. Biomaterials.

[38] Pasini Cabello S.D., Takara E.A., Marchese J., Ochoa N.A. (2015). Influence of plasticizers in pectin films: Microstructural changes. Mater. Chem. Phys..

[39] Hoque M.S., Benjakul S., Prodpran T. (2011). Properties of film from cuttlefish (*Sepia pharaonis*) skin gelatin incorporated with cinnamon, clove and star anise extracts. Food Hydrocoll..

[40] Charulatha V., Rajaram A. (2003). Influence of different crosslinking treatments on the physical properties of collagen membranes. Biomaterials.

[41] Laftah W.A., Hashim S., Ibrahim A.N. (2011). Polymer Hydrogels: A Review. Polym. -Plast. Technol. Eng..

[42] Batista R.A., Espitia P.J.P., Vergne D.M.C., Vicente A.A., Pereira P.A.C., Cerqueira M.A., Teixeira J.A., Jovanovic J., Severino P., Souto E.B. (2020). Development and Evaluation of Superabsorbent Hydrogels Based on Natural Polymers. Polymers.

[43] Zielinska A., Eder P., Rannier L., Cardoso J.C., Severino P., Silva A.M., Souto E.B. (2022). Hydrogels for Modified-release Drug Delivery Systems. Curr. Pharm. Des..

[44] Abruzzo A., Bigucci F., Cerchiara T., Cruciani F., Vitali B., Luppi B. (2012). Mucoadhesive chitosan/gelatin films for buccal delivery of propranolol hydrochloride. Carbohydr. Polym..

[45] Hinrichs W.L.J., Lommen E.J.C.M.P., Wildevuur C.R.H., Feijen J. (1992). Fabrication and characterization of an asymmetric polyurethane membrane for use as a wound dressing. J. Appl. Biomater..

[46] Zorzi Bueno C., Maria Moraes Â. (2011). Development of porous lamellar chitosan-alginate membranes: Effect of different surfactants on biomaterial properties. J. Appl. Polym. Sci..

[47] Rattaya S., Benjakul S., Prodpran T. (2009). Properties of fish skin gelatin film incorporated with seaweed extract. J. Food Eng..

[48] Bodini R.B., Sobral P.J.A., Favaro-Trindade C.S., Carvalho R.A. (2013). Properties of gelatin-based films with added ethanol–propolis extract. LWT-Food Sci. Technol..

[49] Lakra R., Kiran M.S., Usha R., Mohan R., Sundaresan R., Korrapati P.S. (2014). Enhanced stabilization of collagen by furfural. Int. J. Biol. Macromol..

[50] de Menezes A.S., Remédios C.M.R., Sasaki J.M., da Silva L.R.D., Góes J.C., Jardim P.M., Miranda M.A.R. (2007). Sintering of nanoparticles of α-Fe2O3 using gelatin. J. Non-Cryst. Solids.

[51] Jalaja K., Naskar D., Kundu S.C., James N.R. (2015). Fabrication of cationized gelatin nanofibers by electrospinning for tissue regeneration. RSC Adv..

[52] Yakimets I., Wellner N., Smith A.C., Wilson R.H., Farhat I., Mitchell J. (2005). Mechanical properties with respect to water content of gelatin films in glassy state. Polymer.

[53] Dashnyam K., Perez R., Lee E.-J., Yun Y.-R., Jang J.-H., Wall I.B., Kim H.-W. (2014). Hybrid scaffolds of gelatin–siloxane releasing stromal derived factor-1 effective for cell recruitment. J. Biomed. Mater. Res. A.

[54] de Macedo L.M., Santos É.M.d., Militão L., Tundisi L.L., Ataide J.A., Souto E.B., Mazzola P.G. (2020). Rosemary (*Rosmarinus officinalis* L., syn *Salvia rosmarinus* Spenn.) and Its Topical Applications: A Review. Plants.

[55] Ergene Öz B., Saltan İşcan G., Küpeli Akkol E., Süntar İ., Keleş H., Bahadır Acıkara Ö. (2017). Wound healing and anti-inflammatory activity of some Ononis taxons. Biomed. Pharmacother. Biomed. Pharmacother..

[56] Henriques B.O., Corrêa O., Azevedo E.P., Pádua R.M., de Oliveira V.L., Oliveira T.H., Boff D., Dias A.C., de Souza D.G., Amaral F.A. (2016). In Vitro TNF-α Inhibitory Activity of Brazilian Plants and Anti-Inflammatory Effect of Stryphnodendron adstringens in an Acute Arthritis Model. Evid. -Based Complement. Altern. Med. Ecam.

[57] Sánchez-Fidalgo S., da Silva M.S., Cárdeno A., Aparicio-Soto M., Salvador M.J., Frankland Sawaya A.C., Souza-Brito A.R., de la Lastra C.A. (2013). Abarema cochliacarpos reduces LPS-induced inflammatory response in murine peritoneal macrophages regulating ROS-MAPK signal pathway. J. Ethnopharmacol..

[58] Su W.H., Cheng M.H., Lee W.L., Tsou T.S., Chang W.H., Chen C.S., Wang P.H. (2010). Nonsteroidal anti-inflammatory drugs for wounds: Pain relief or excessive scar formation?. Mediat. Inflamm..

[59] Wang P.H., Huang B.S., Horng H.C., Yeh C.C., Chen Y.J. (2018). Wound healing. J. Chin. Med. Assoc. JCMA.

[60] Shurtz-Swirski R., Sela S., Herskovits A.T., Shasha S.M., Shapiro G., Nasser L., Kristal B. (2001). Involvement of peripheral polymorphonuclear leukocytes in oxidative stress and inflammation in type 2 diabetic patients. Diabetes Care.

[61] Sela S., Shurtz-Swirski R., Awad J., Shapiro G., Nasser L., Shasha S.M., Kristal B. (2002). The involvement of peripheral polymorphonuclear leukocytes in the oxidative stress and inflammation among cigarette smokers. Isr. Med. Assoc. J. IMAJ.

[62] Kapoor M., Howard R., Hall I., Appleton I. (2004). Effects of epicatechin gallate on wound healing and scar formation in a full thickness incisional wound healing model in rats. Am. J. Pathol..

[63] Kim H., Kawazoe T., Han D.W., Matsumara K., Suzuki S., Tsutsumi S., Hyon S.H. (2008). Enhanced wound healing by an epigallocatechin gallate-incorporated collagen sponge in diabetic mice. Wound Repair Regen. Off. Publ. Wound Heal. Soc. Eur. Tissue Repair Soc..

[64] Klass B.R., Branford O.A., Grobbelaar A.O., Rolfe K.J. (2010). The effect of epigallocatechin-3-gallate, a constituent of green tea, on transforming growth factor-beta1-stimulated wound contraction. Wound Repair Regen. Off. Publ. Wound Heal. Soc. Eur. Tissue Repair Soc..

[65] Hajiaghaalipour F., Kanthimathi M.S., Abdulla M.A., Sanusi J. (2013). The Effect of Camellia sinensis on Wound Healing Potential in an Animal Model. Evid. -Based Complement. Altern. Med. Ecam.

[66] Schmidt C.A., Murillo R., Bruhn T., Bringmann G., Goettert M., Heinzmann B., Brecht V., Laufer S.A., Merfort I. (2010). Catechin derivatives from Parapiptadenia rigida with in vitro wound-healing properties. J. Nat. Prod..

[67] Gantwerker E.A., Hom D.B. (2012). Skin: Histology and physiology of wound healing. Clin. Plast. Surg..

[68] Tanigawa T., Kanazawa S., Ichibori R., Fujiwara T., Magome T., Shingaki K., Miyata S., Hata Y., Tomita K., Matsuda K. (2014). (+)-Catechin protects dermal fibroblasts against oxidative stress-induced apoptosis. BMC Complement. Altern. Med..

[69] Silva Santos L.F., Stolfo A., Calloni C., Salvador M. (2017). Catechin and epicatechin reduce mitochondrial dysfunction and oxidative stress induced by amiodarone in human lung fibroblasts. J. Arrhythmia.

[70] Morin M.P., Grenier D. (2017). Regulation of matrix metalloproteinase secretion by green tea catechins in a three-dimensional co-culture model of macrophages and gingival fibroblasts. Arch. Oral Biol..

